# Supplementary material for: Monolithically-grained perovskite solar cell with Mortise-Tenon structure for charge extraction balance
Source: Nat Commun. 2023 Jun 3;14:3216. doi: 10.1038/s41467-023-38926-3 (PMC10239504; doi:10.1038/s41467-023-38926-3)
Supplement: Supplementary file 1 — Supplementary Information [file 41467_2023_38926_MOESM1_ESM.pdf]

Supplementary Information

Monolithically-Grained Perovskite Solar Cell with Mortise-Tenon Structure for Charge Extraction Balance

Wang et al.

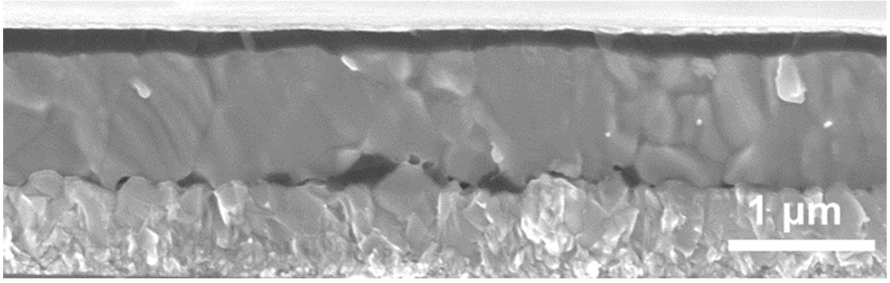

Supplementary Fig. 1. The cross-sectional SEM image of the control film.

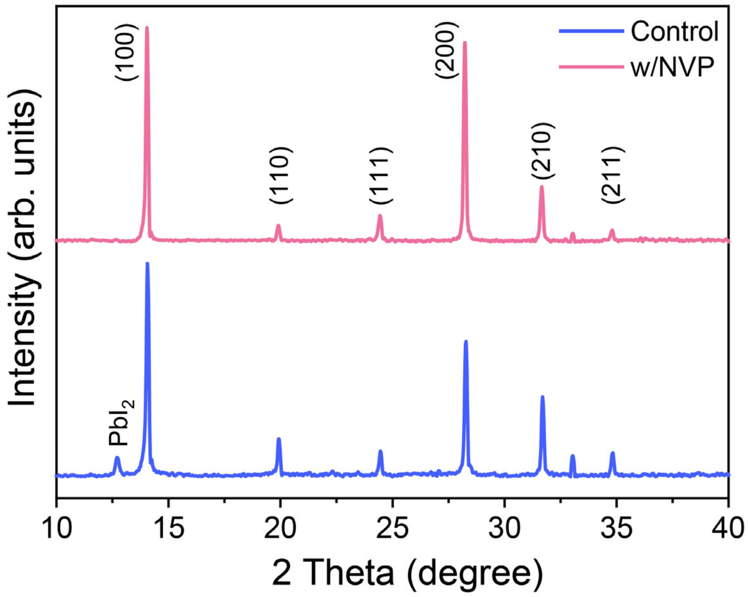

Supplementary Fig. 2. X-ray diffraction patterns of control and perovskite/NVP films.

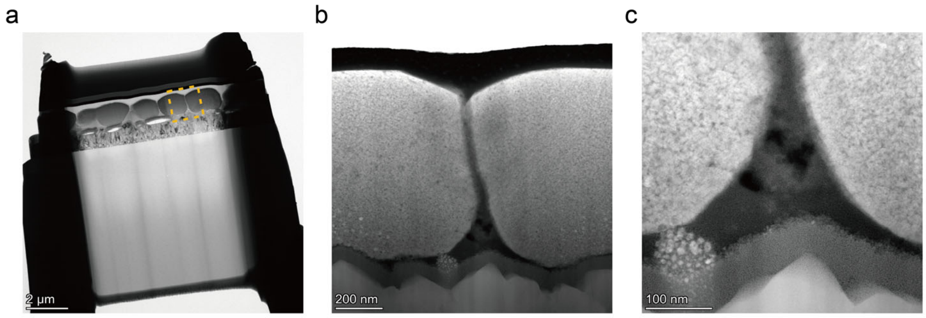

Supplementary Fig. 3. Low magnification **a** and **b**, and high magnification **c** HAADF image of NVP-based PSCs based on $(FAPbI_3)_{0.95}(MAPbBr_3)_{0.05}$ perovskite and spiro-OMeTAD as HTL.

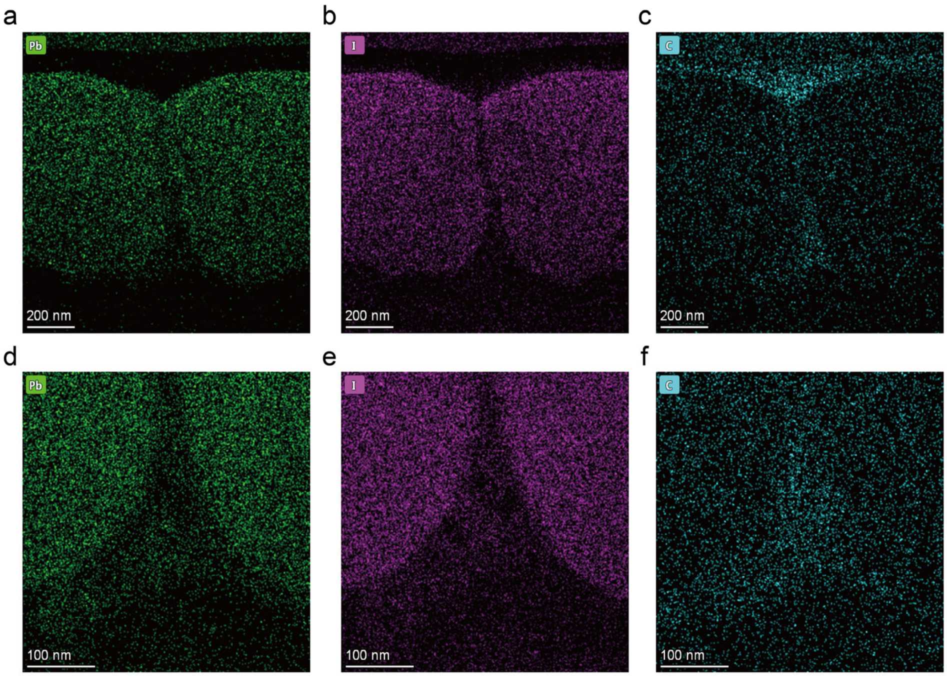

Supplementary Fig. 4. STEM-EDS elemental mapping of **a** and **d,** Pb, **b** and **e,** I, **c** and **f,** C in NVP-based PSCs based on $(FAPbI_3)_{0.95}(MAPbBr_3)_{0.05}$ perovskite and spiro-OMeTAD as HTL.

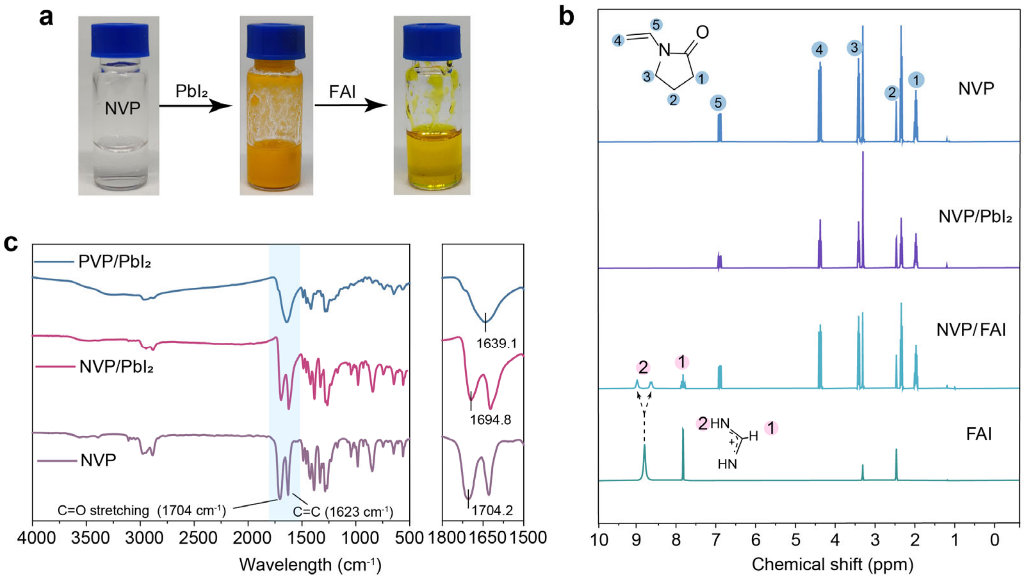

Supplementary Fig. 5. **a**, Photos of pure NVP, NVP/PbI_2 mixture, and NVP/PbI_2/FAI mixture. **b**, comparison of ^{1}H NMR spectra of NVP, NVP/PbI_2, NVP/FAI/ PbI_2, and FAI. **c**. FTIR spectra of the NVP, NVP/PbI_2, and PVP/PbI_2 (NVP/PbI_2 film annealing 100 °C for 1h).

Supplementary Discussion 1.

For directly investigate the interaction of NVP and perovskite precursors, as shown in Supplementary Fig. 5a, PbI_2 was added into the pure NVP, leading to a formation of yellow precipitation, then after adding FAI into this mixture, the precipitation disappeared and the solution became clarified. This observation suggested a synergistic interaction between NVP, FAI and PbI_2. ^{1}H NMR spectra as shown in Supplementary Fig. 5b also proved this speculation. With the addition of FAI in NVP, the single resonance peak (8.788 ppm) of the ammonium in FAI spilt into two at 8.973 ppm and 8.608 ppm, implying that the strong interaction between FA^+ and NVP. The variation of ^{1}H NMR spectra of the NVP/ PbI_2 solution and NVP is minimal, indicating that the interaction between NVP and PbI_2 in solution is negligible. However, this changed substantially in the case of solid films. As shown in Supplementary Fig. 5c, Fourier Transform Infrared Spectroscopy (FTIR) confirmed that C=O of NVP could strongly coordinate with Pb^{2+}. For pure NVP, the stretching vibration of the C=O bond at 1704 cm^{-1} shifted to lower wavenumber at 1694 cm^{-1} after addition of PbI_2, and further shifted to 1639 cm^{-1} after NVP annealing to PVP polymer. The apparent shift of the C=O absorption peak indicated the strong coordination between NVP and Pb^{2+} in the solid film state.

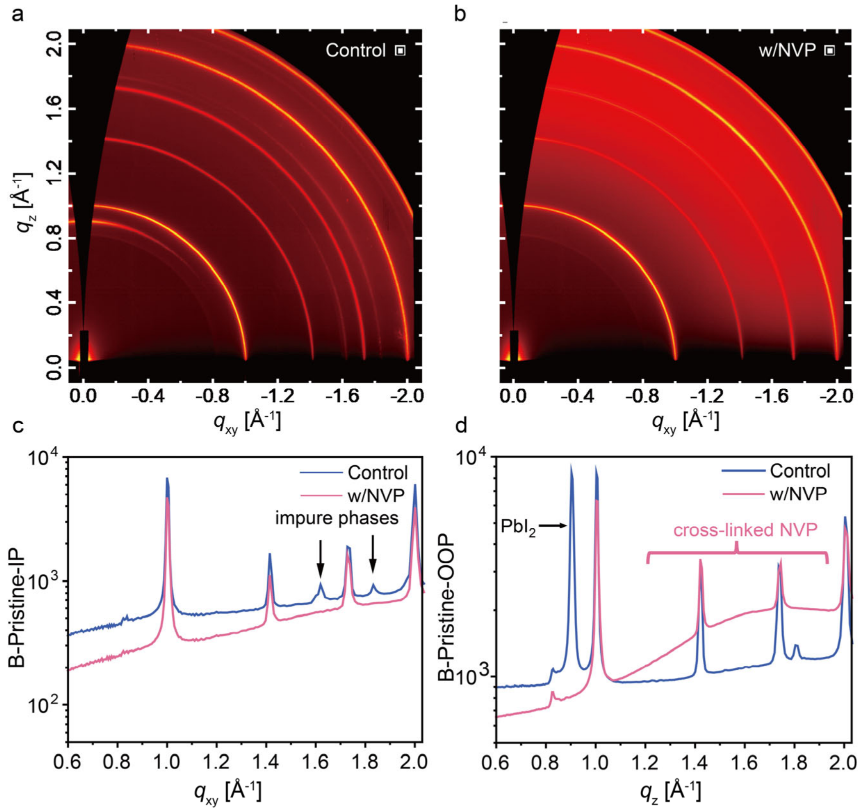

Supplementary Fig. 6. GIWAXS patterns of **a,** control perovskite and **b,** perovskite/NVP films. In-plane and out-of-plane 1D plots of **c,** control perovskite and **d,** perovskite/NVP films.

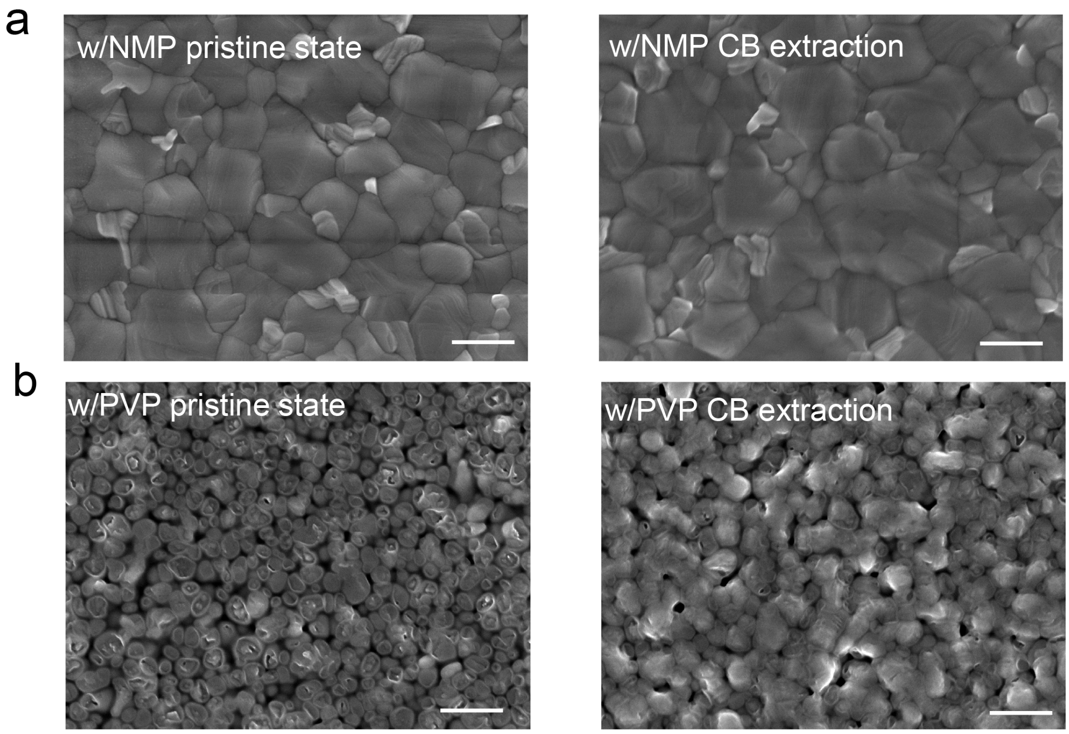

Supplementary Fig. 7 Surface SEM images of **a,** the perovskite/NMP films and **b,** perovskite/PVP film before and after CB extraction, the scale bar is 1μm.

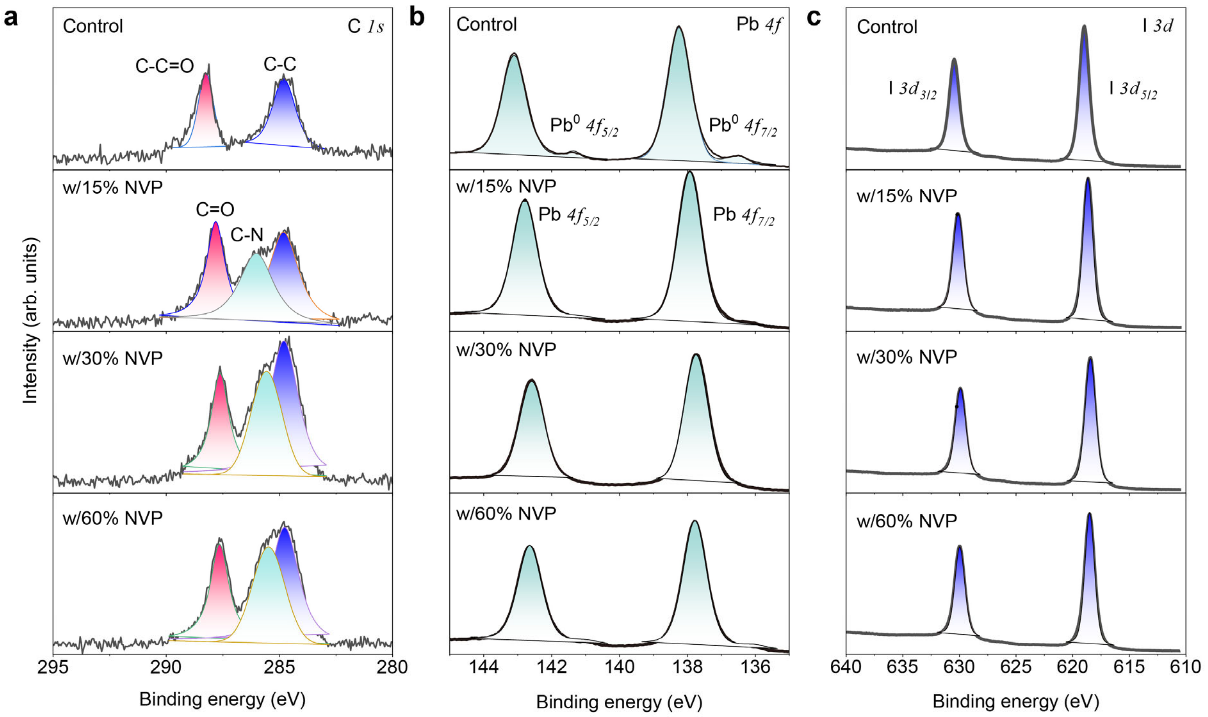

Supplementary Fig. 8. XPS spectra of **a,** C, **b,** Pb, and **c,** I signal in perovskite films with different molar ratios of NVP.

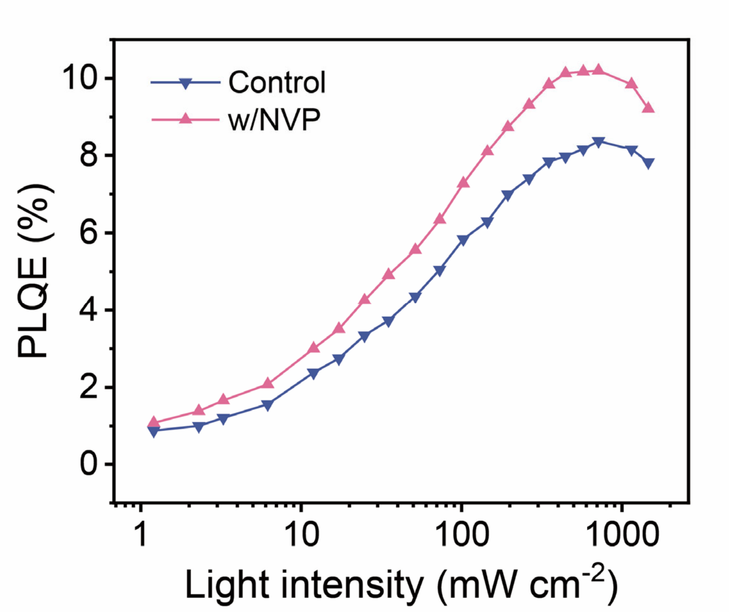

Supplementary Fig. 9. Excitation-intensity-dependent PLQE of control perovskite and perovskite/NVP films.

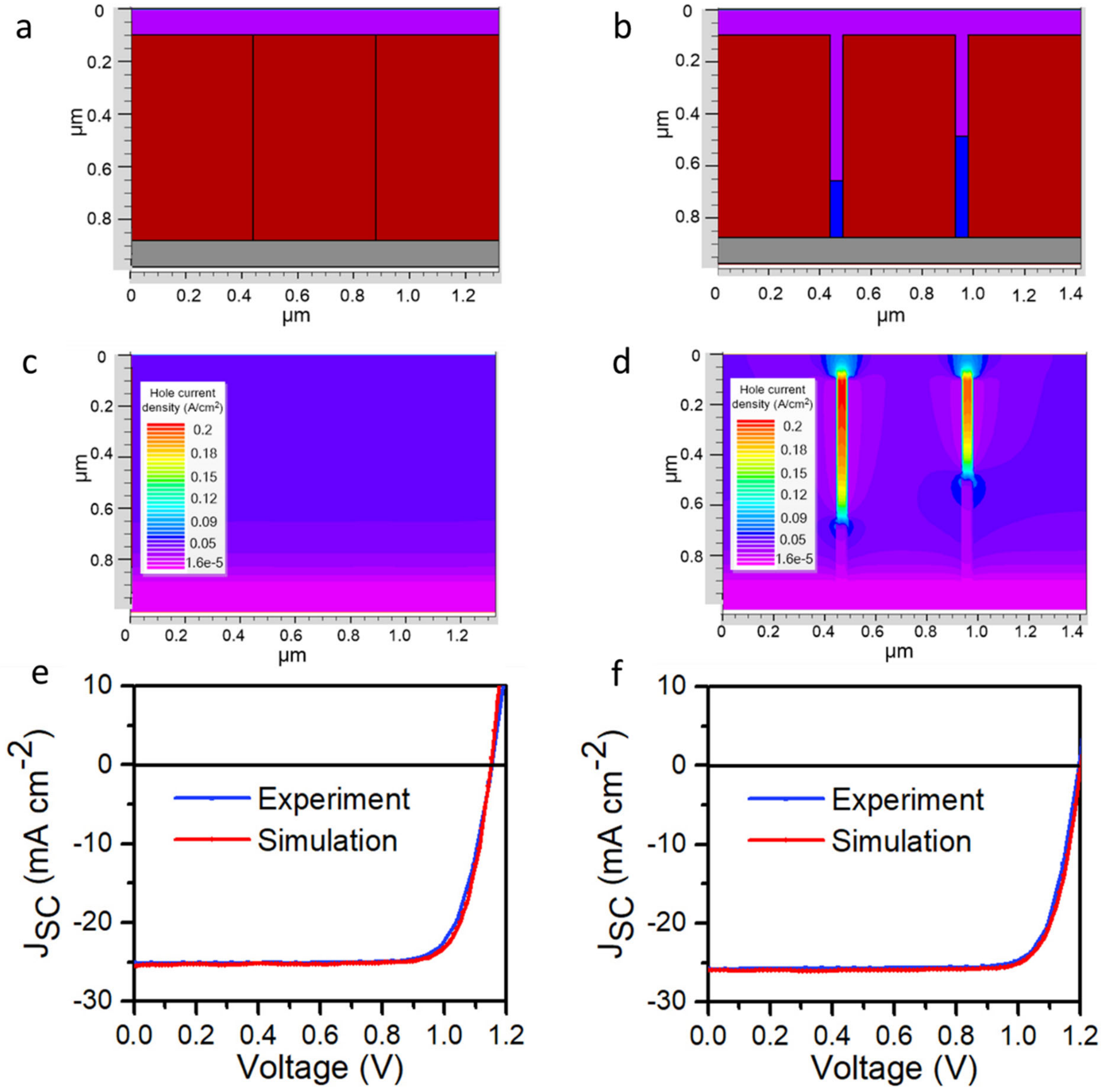

Supplementary Fig. 10. Schematic diagrams of **a,** control, and **b**, NVP model with M-T structure. Hole current density distribution of the simulated **c,** control, and **d,** NVP-based PSCs. The simulated perovskite grain size was 780 nm. GB grooves were assumed to be 390 nm and 560 nm deep, which was consistent with the STEM measurements. Corresponding experimental or theoretical *J*–*V* curves of **e,** control and **f,** NVP-based PSCs.

Supplementary Table 1. Experimental or theoretical performances of control and NVP-based PSCs

architecture	data type	$\boldsymbol{V_{OC}}$ [V]	$\boldsymbol{J_{SC}}$ [mA cm^{-2}]	**FF** [%]	**PCE** [%]
control	experimental	1.151	25.21	78.94	22.91
	Simulation	1.15	25.46	79.15	23.17
w/NVP	experimental	1.195	25.77	80.19	24.69
	Simulation	1.20	25.96	80.89	25.19

Device simulation: Silvaco software was used for device simulation with structure of FTO/SnO_2/perovskite/spiro-OMeTAD/Au. The DevEdit module was used to construct the device structure. A narrow region with thickness of 1 nm was created between two adjacent perovskite grains to simulate grain boundary. The grain boundary region was assumed to be p-type doping with doping level of 10^{15} cm^{-3}. The recombination of carriers was considered by calculating Shockley-Read-Hall (SRH) and Auger recombination. Tonyplot was used to get the results from simulation.

Supplementary Table 2. Parameters of the materials for device simulation[1,2].

**Parameters and units**	**Symbol**	**SnO_2**	**PSK**	**spiro-OMeTAD**
Thickness (nm)	L	100	780	100
Band Gap at 300 K (eV)	E_g	3.8	1.55	3.0
Electron Affinity (eV)	χ	4.31	4.22	2.3
Electron density of states at 300 K (#/cm^3)	N_C	1×10^{21}	2.5×10^{20}	2.5×10^{20}
Hole density of states at 300 K (#/cm^3)	N_V	2×10^{20}	2.5×10^{20}	2.5×10^{20}
Hole mobility (cm^2 V^{-1} s^{-1})	μ_h		1.0	0.001
Electron mobility (cm^2 V^{-1} s^{-1})	μ_e	10	1.0	
Relative dielectric constant	ε_r	100	46.9	3
Doping level (#/cm^3)	N_D	2.5×10^{17} (n-type)	-	2.5×10^{17} (p-type)

Supplementary Table 3. Parameters of the time-correlated single photon counting (TCSPC) spectroscopy of perovskite films (glass/FTO/SnO_2/$(FAPbI_3)_{0.95}(MAPbBr_3)_{0.05}$ PSK/spiro-OMeTAD) from ETL (SnO_2) side and HTL (spiro-OMeTAD) side, respectively.

**PSK**	**side**	τ_1**[ns]**	A_1**[%]**	τ_2**[ns]**	A_2**[%]**	τ_{ave}**[ns]**
Control	ETL	0.3581	36.42	1.50848	63.58	1.3707
w/NVP	ETL	0.3328	32.81	1.67520	67.19	1.2347
Control	HTL	0.7721	2.060	22.5708	97.94	22.4151
w/NVP	HTL	0.3797	45.51	1.7183	54.49	1.5097

The PL decay time and amplitudes are modeled using a biexponential expression [3]:

$$f(t) = \sum_i A_i \, exp\left(-\frac{t}{\tau_i}\right) + K$$

where A_i is the decay amplitude, τ_i is the decay time and K is a constant for the base-line offset. The average PL decay times (τ_{ave}) are further estimated using the τ_i and A_i values from the fitted curve data Table S1 using:

$$\tau_{ave} = \frac{\sum A_i \tau_i^2}{\sum A_i \, \tau_i}$$

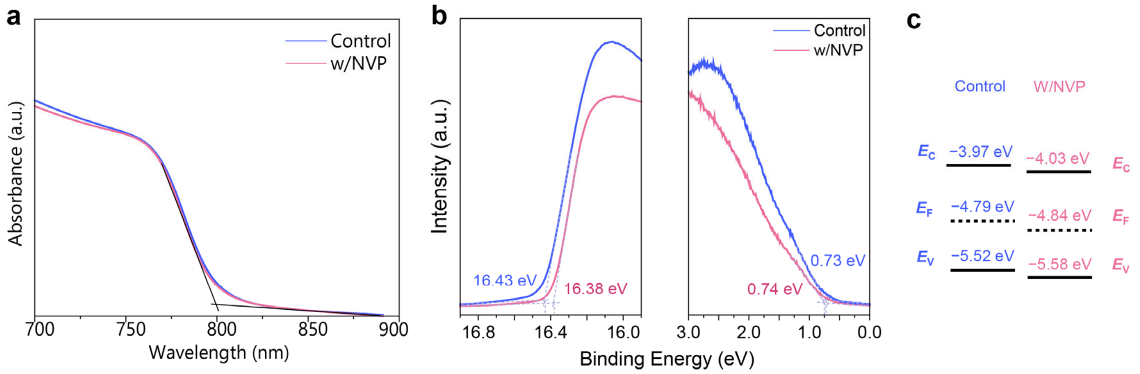

Supplementary Fig. 11. a, UV-vis absorption spectra of the control and perovskite/NVP film. **b,** Valence-band region and photoemission cut off energy of the UPS spectra. **c**, Energy-level diagram constructed from UPS results. Conduction band minimum (E_C), Valence band maximum (E_V), and Fermi level (E_F).

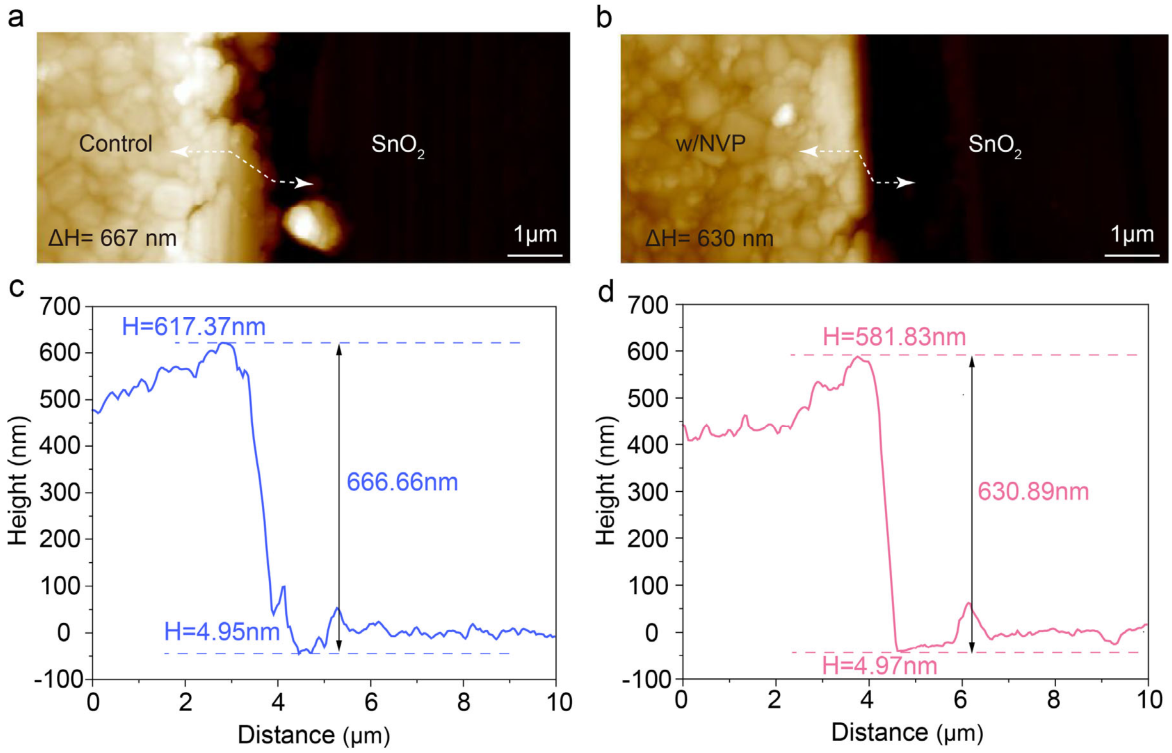

Supplementary Fig. 12. Surface height images of **a,** Control and **b,** perovskite/NVP films. Corresponding surface height of the **c**, control, and **d,** perovskite/NVP films.

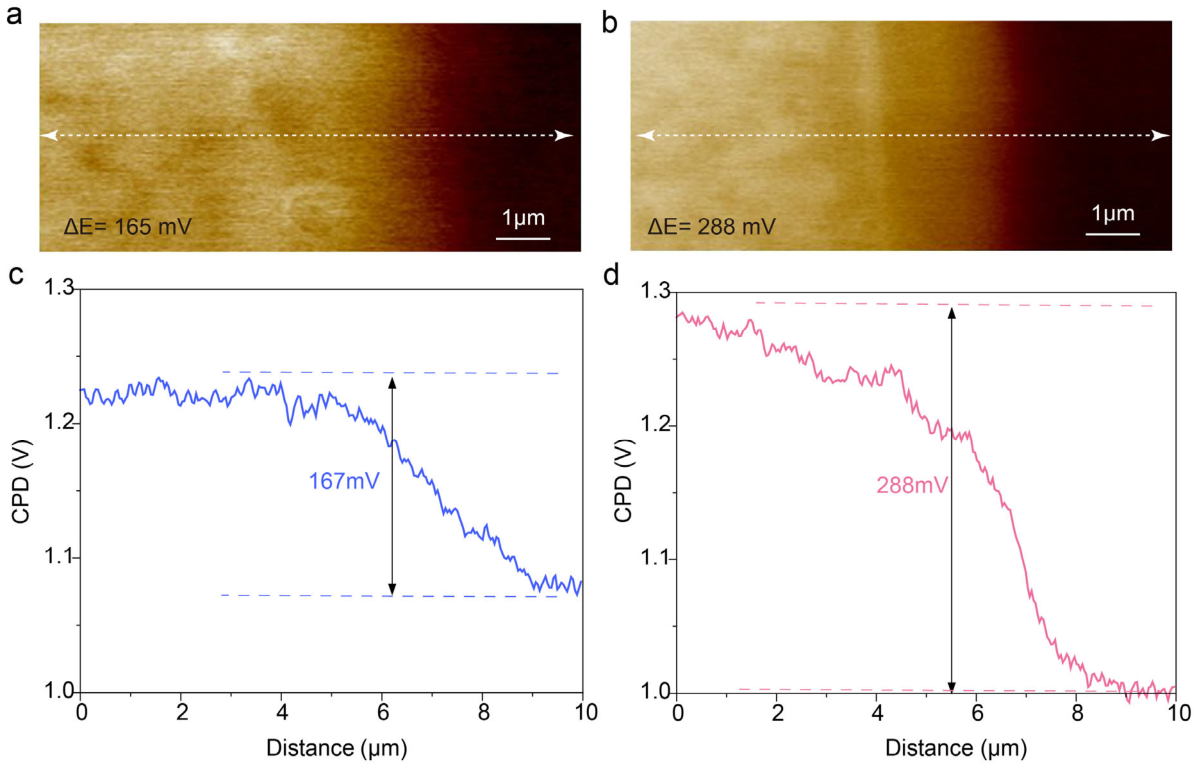

Supplementary Fig. 13. KPFM images of the interface between the perovskite and SnO_2 layers. **a,** Control and **b,** perovskite/NVP films. Corresponding values of surface contact potential difference (CPD) of **c**, control, and **d,** perovskite/NVP films.

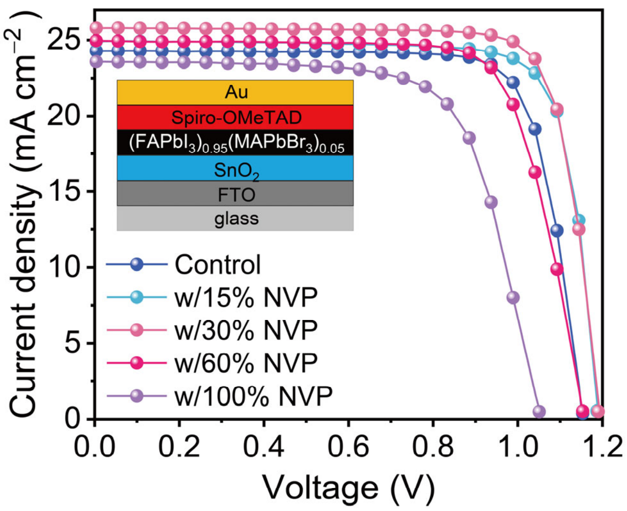

Supplementary Fig. 14. Current density-voltage (*J-V*) curves of PSCs based on perovskite with different molar ratios of NVP.

Supplementary Table 4. Device performance of PSCs with different molar ratios of NVP.

**NVP** [mol%]	V_{OC} [V]	J_{SC} [mA cm^{-2}]	**FF** [%]	**PCE** [%]
Control	1.151	25.21	78.94	22.91
w/NVP (15%)	1.191	24.91	80.05	23.75
w/NVP (30%)	1.195	25.77	80.19	24.69
w/NVP (60%)	1.157	24.95	75.26	21.73
w/NVP (100%)	1.052	23.56	69.80	17.32

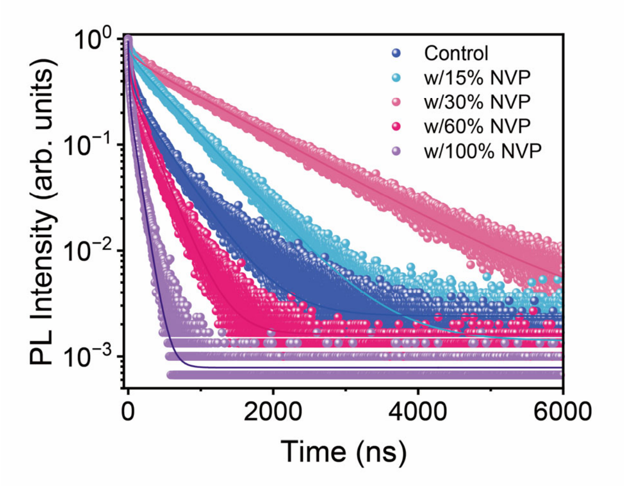

Supplementary Fig. 15. Time-resolved photoluminescence decay curves (excitation: 520nm, 2.26 nJ cm^{-2}, 0.1 MHz).

Supplementary Table 5. TCSPC fitting lifetimes of perovskite films with different molar ratios of NVP.

PSK	τ_1[ns]	A_1[%]	τ_2[ns]	A_2[%]	τ_{ave}[ns]
Control	56.54	11.66	471.81	88.34	375.69
15%	74.93	2.4	581.50	97.6	569.34
30%	114.8	2.19	1160.73	97.81	1138.42
60%	27.03	9.23	252.82	90.77	231.97
100%	11.26	21.41	95.75	78.59	77.66

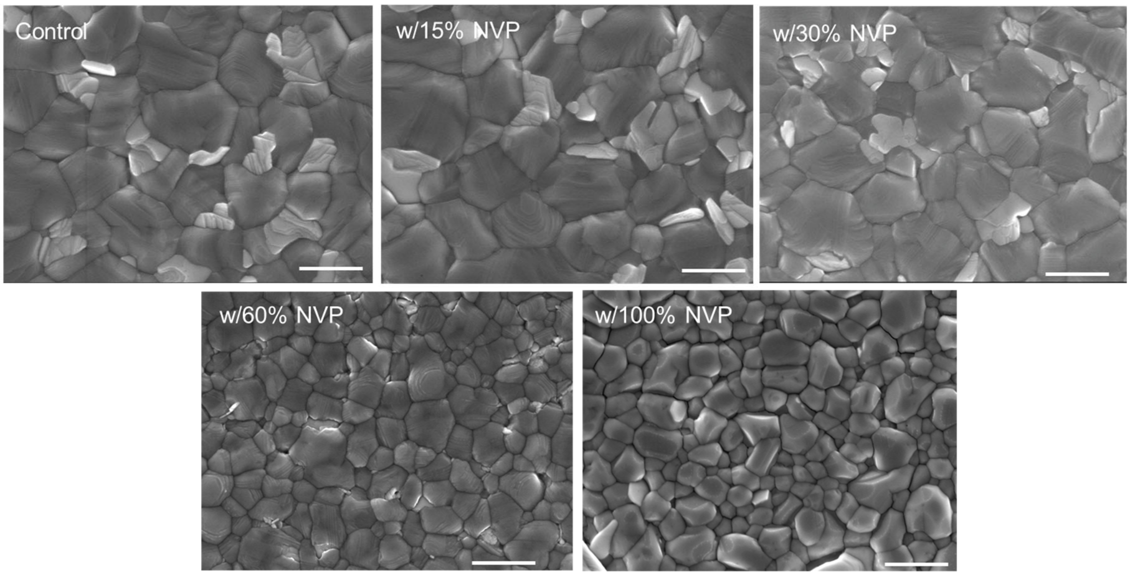

Supplementary Fig.16. Surface SEM images of perovskite films with different molar ratios of NVP, the scale bar is 1μm.

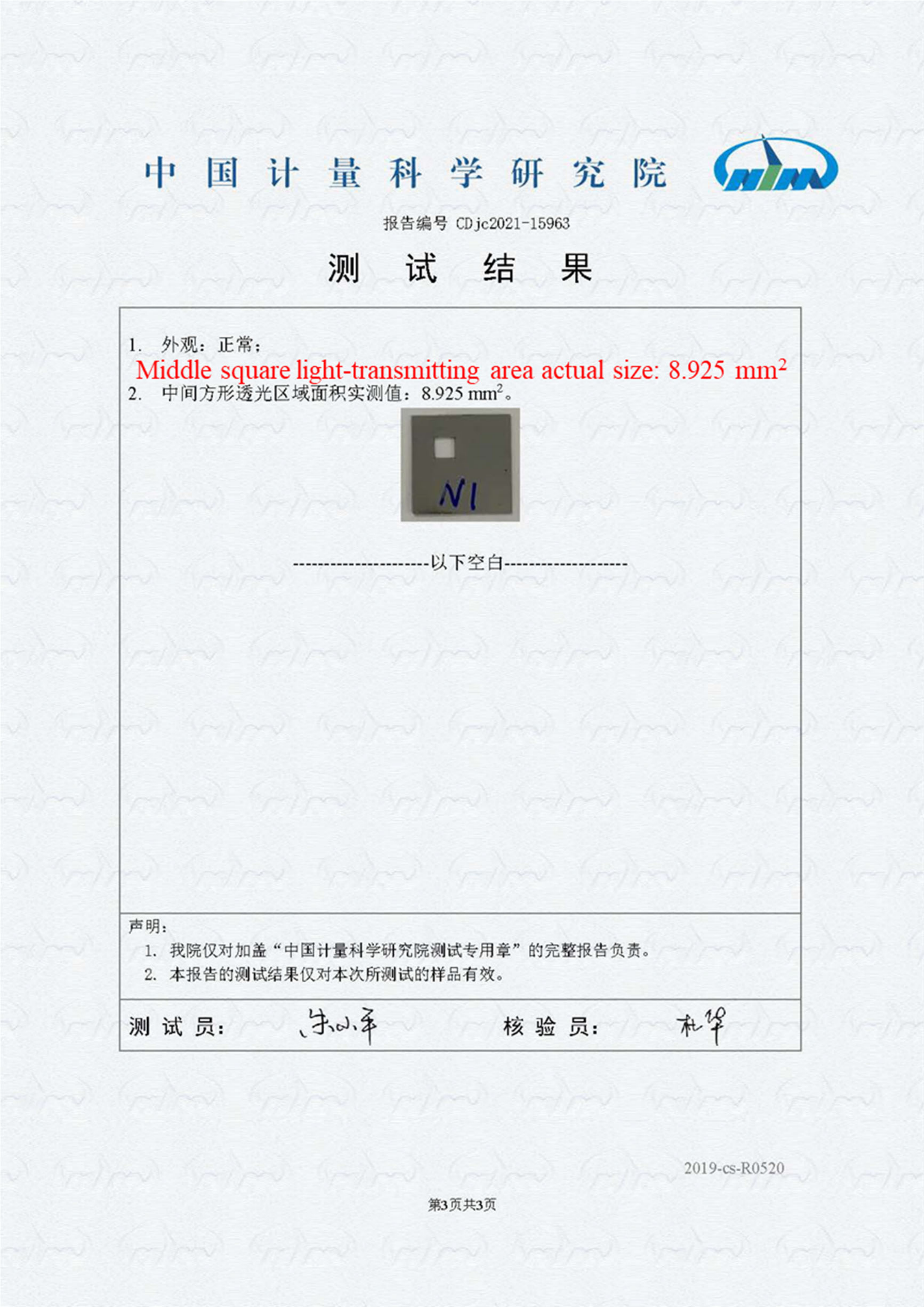

中 国 计 量 科 学 研 究 院

报告编号 CDjc2021-15963

测 试 结 果

1. 外观：正常；

Middle square light-transmitting area actual size: 8.925 mm^2

2. 中间方形透光区域面积实测值：8.925 mm^2。

N1

---------------------以下空白-------------------

声明：

1. 我院仅对加盖“中国计量科学研究院测试专用章”的完整报告负责。
2. 本报告的测试结果仅对本次所测试的样品有效。

测 试 员：　　　　核 验 员：

2019-cs-R0520

第3页共3页

Supplementary Fig. 17. Certification report on the size of the middle square light-transmitting area provided by National institute of metrology, China. (Test report No. CDjc2021-15963)

中国认可
检测
TESTING
CNAS L2338

TEST REPORT

Report No：PWQC-WT-P21110821-1R

**Sample Name**	:	Photovoltaic cell
**Client**	:	Nanjing Tech University<br>Institute of Advanced Materials
**Client Address**	:	5# XinMoFan Road, Nanjing, Jiangsu
**Type of Project**	:	Consignation

检测专用章

PHOTOVOLTAIC AND WIND POWER SYSTEMS QUALITY TEST CENTER，IEE, CHINESE ACADEMY OF SCIENCES

November, 11, 2021

PHOTOVOLTAIC AND WIND POWER SYSTEMS QUALITY TEST CENTER, IEE, CHINESE ACADEMY OF SCIENCES

Report No: PWQC-WT-P21110821-1R

Testing information:

Date：November, 8, 2021

Location：No.6 Bei-er-tiao, Zhongguancun, Haidian district, Beijing, China

Environmental conditions：24℃, 36.5%RH

Testing items：

Measurement of photovoltaic current-voltage characteristics

Standards：

IEC 60904-1：2006 Photovoltaic (PV) devices
— Part 1：Measurement of photovoltaic current-voltage characteristics

Equipments：

Name	S/N	Expired date
Solar simulator	LE106-04	2022-05-13
Source Meter	LE177-01	2022-03-02
Digital millimeter	LE126-01	2022-02-21
Reference cell	J-CH02	2022-08-26

Edited by(signatory)：Yang Lei
Date：2021.11.11

Approved by(signatory)：Jiang Feifei
Date：2021.11.11

PHOTOVOLTAIC AND WIND POWER SYSTEMS QUALITY TEST CENTER, IEE, CHINESE ACADEMY OF SCIENCES

Report No: PWQC-WT-P21110821-1R

**Sample code**	DC2021a031
**Sample S/N**	1
**Type**	Single junction perovskite
**Sample description**	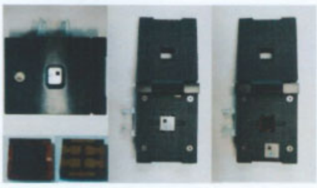
**Designated area**	$8.925mm^2$<br>The designated area was provided by National Institute of Metrology, China. Test Report No. CDjc2021-15963.

**Items of testing**	Measurement of photovoltaic current-voltage characteristics					
**Sample code**	DC2021a031					
**Results**	**Voltage Sweep**	**Isc** (mA)	**Jsc** (mA/cm^2)	**Voc** (V)	**Pm** (mW)	**File**
	**Forward**	2.268	25.41	1.188	2.165	A202111081 42544
		**Ipm** (mA)	**Vpm** (V)	**FF** (%)	**E_{ff}** (%)	
		2.165	1.000	80.35	24.25	
	**Voltage Sweep**	**Isc** (mA)	**Jsc** (mA/cm^2)	**Voc** (V)	**Pm** (mW)	**File**
	**Reverse**	2.290	25.66	1.187	2.191	A202111081 42200
		**Ipm** (mA)	**Vpm** (V)	**FF** (%)	**E_{ff}** (%)	
		2.191	1.000	80.64	24.55	

Measurement uncertainty:

$U_{95(Isc)}$=1.9% (k=2)

$U_{95(Voc)}$=1.8% (k=2)

$U_{95(Pm)}$=2.5% (k=2)

Page 2 of 4

PHOTOVOLTAIC AND WIND POWER SYSTEMS QUALITY TEST CENTER, IEE, CHINESE ACADEMY OF SCIENCES

Report No: PWQC-WT-P21110821-1R

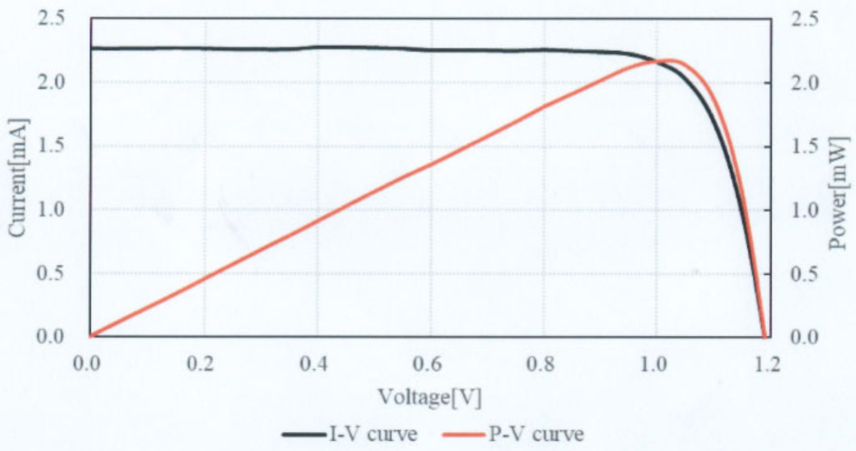

**Type**	Single junction perovskite
**Ser.No**	DC2021a031
**Area**	8.925 mm^2
**Isc**	2.268 mA
**Jsc**	25.41 mA/cm^2
**Voc**	1.188 V
**FF**	80.35 %
**Pm**	2.165 mW
**E_{ff}**	24.25 %
**Ipm**	2.165 mA
**Vpm**	1.000 V
**Voltage Sweep**	Forward
**Sweep time**	2.9 s
**Temp**	25 ℃
**Irr**	100 mW/cm^2
**File**	A20211108142544

Page 3 of 4

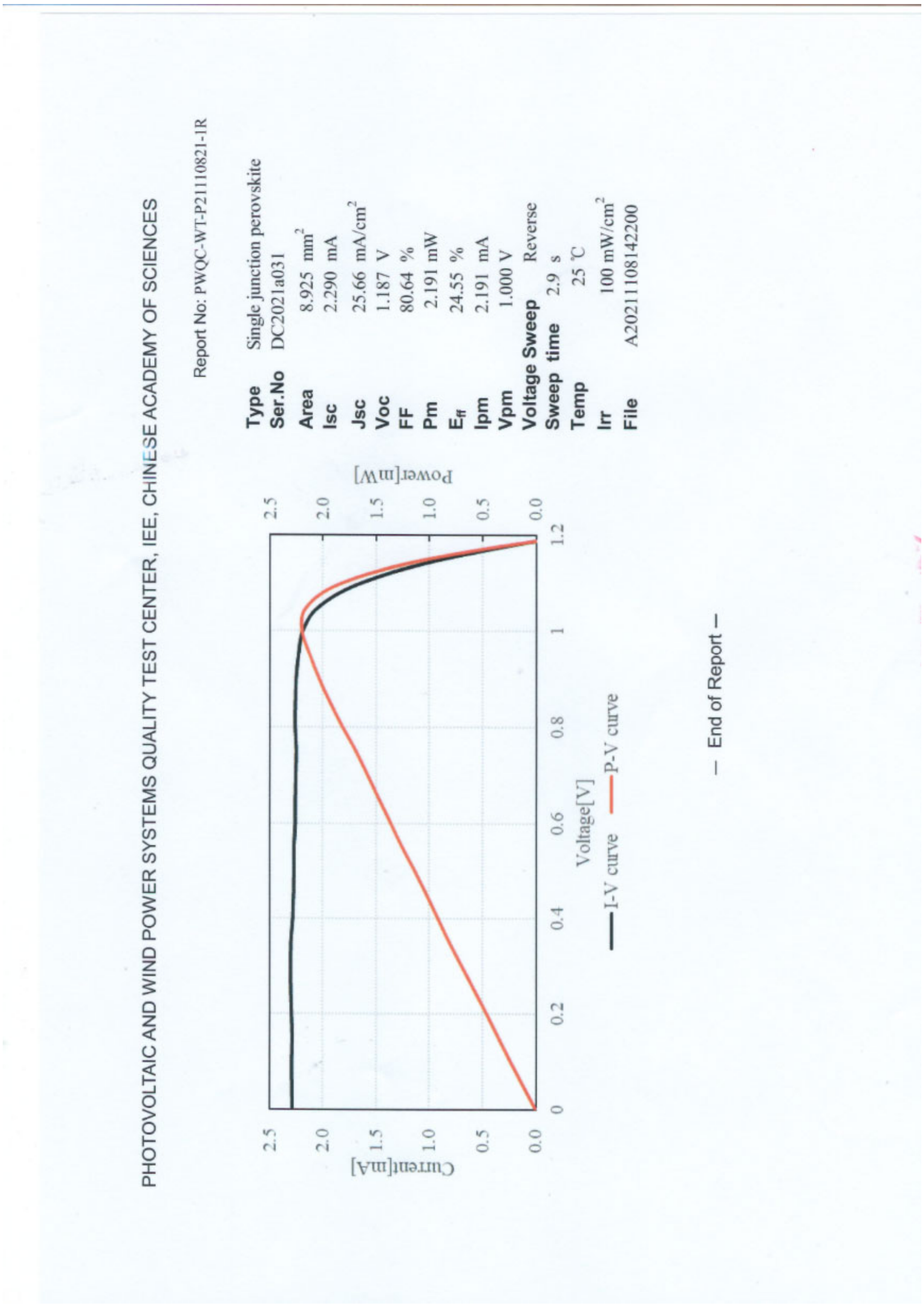
PHOTOVOLTAIC AND WIND POWER SYSTEMS QUALITY TEST CENTER, IEE, CHINESE ACADEMY OF SCIENCES

Report No: PWQC-WT-P21110821-1R

Type	Single junction perovskite
Ser.No	DC2021a031
Area	8.925 mm^2
Isc	2.290 mA
Jsc	25.66 mA/cm^2
Voc	1.187 V
FF	80.64 %
Pm	2.191 mW
E_{ff}	24.55 %
Ipm	2.191 mA
Vpm	1.000 V
Voltage Sweep	Reverse
Sweep time	2.9 s
Temp	25 ℃
Irr	100 mW/cm^2
File	A20211108142200

— End of Report —

Supplementary Fig. 18. Certification report provided by Photovoltaic and wind power systems quality test center, Chinese academy of sciences for certification. (Test report No. PWQC-WT-P21110821-1R)

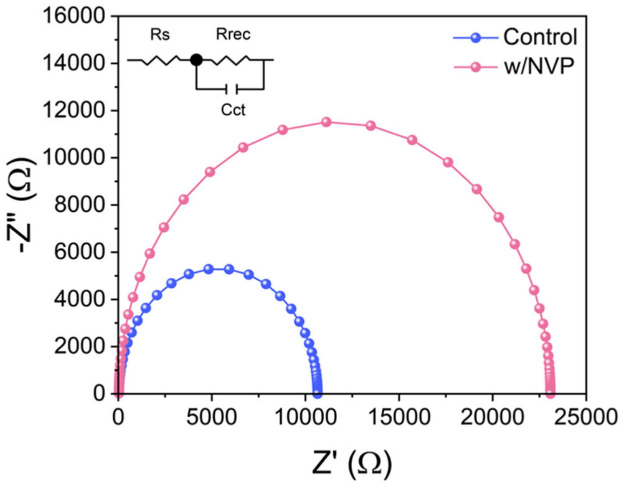

Supplementary Fig. 19. Impedance image and the corresponding equivalent circuits of the Control and NVP-based PSCs**.**

Supplementary Table 6. Fitted values of different electronic parameters from dark Nyquist plots of control and NVP-based PSCs.

	R_s (Ω)	R_{rec} (Ω)	C_{ct} (nF)
Control	87.58	10490	63.04
w/NVP	41.67	22810	60.68

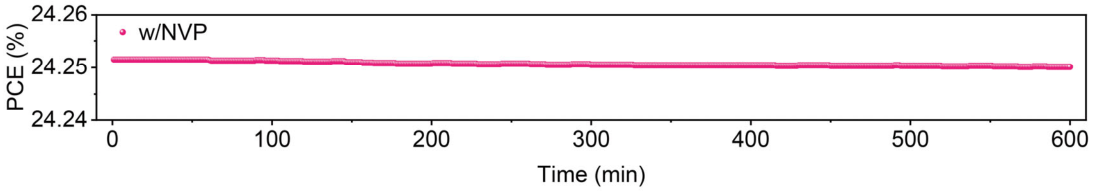

Supplementary Fig. 20. Steady power outputs by tracking the NVP-based PSC at maximum power point.

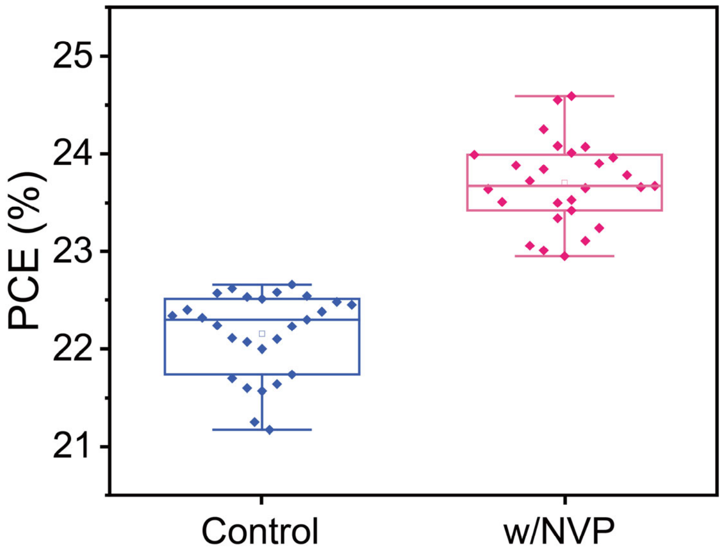

Supplementary Fig. 21. Box charts illustrating the statistical distribution of the Control and NVP-based PSCs for best-performing devices. Each group consists of 27 devices.

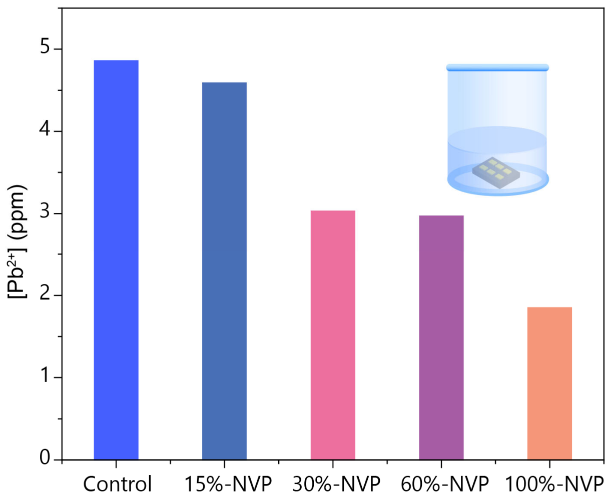

Supplementary Fig. 22. The lead ion concentration in the aqueous solution after perovskite films soaking in water with different molar ratios of NVP for 1 hour. The lead ion concentration in water was determined by atomic absorption spectrophotometer (AAS) measurement.

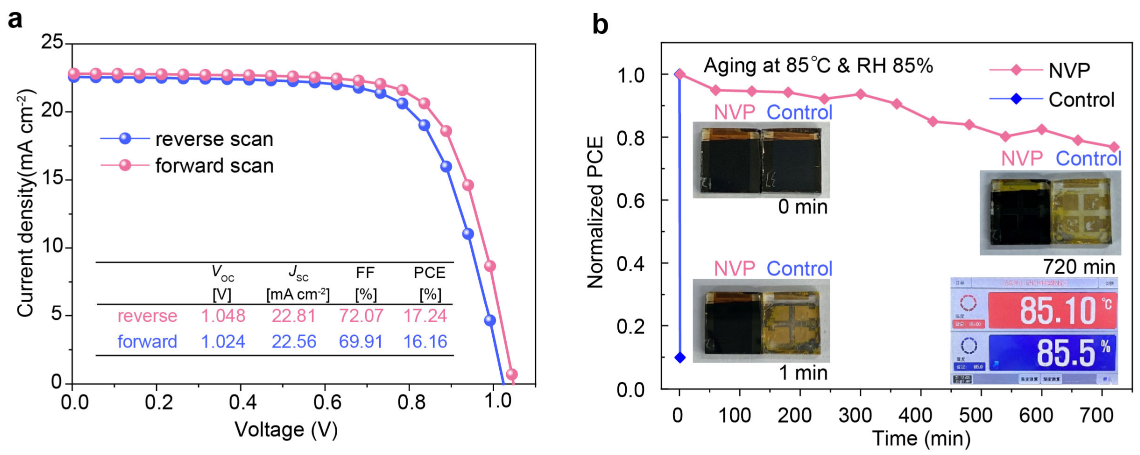

Supplementary Fig. 23. a, *J-V* curves measured at reverse and forward scan of 1.474 M $(FAPbI_3)_{0.95}(MAPbBr_3)_{0.05}$ precursor was dissolved in 1 mL of pristine NVP solvent PSCs. b, Tracking of environmental stability of devices in an environment (85 °C, 85% RH) for 720 min and the images of unencapsulated devices.

Supplementary References

[1] P. You et al., *Light Sci. Appl.* **10**, 68 (2021).

[2] I.G. Tamizhmani et al., *Appl. Phys. A* **127**, 923 (2021).

[3] F.-Z. Qiu et al., *J. Mater. Chem. A* **9**, 24064-24070 (2021).
