# Supplementary material for: Monolithically-grained perovskite solar cell with Mortise-Tenon structure for charge extraction balance
Source: Nat Commun. 2023 Jun 3;14:3216. doi: 10.1038/s41467-023-38926-3 (PMC10239504; doi:10.1038/s41467-023-38926-3)
Supplement: Supplementary file 3 — Description of Additional Supplementary Files [file 41467_2023_38926_MOESM3_ESM.pdf]

Description of Additional Supplementary Files

File name: Supplementary Movie 1

Description: Unencapsulated control and perovskite/NVP films in Water-vapor test (100% RH and 100°C). We fumigated the unencapsulated control and perovskite/pure NVP films in hot water vapor and could see that the control films rapidly turned yellow while the NVP films remained in the black phase.
