# Supplementary material for: Monolithically-grained perovskite solar cell with Mortise-Tenon structure for charge extraction balance
Source: Nat Commun. 2023 Jun 3;14:3216. doi: 10.1038/s41467-023-38926-3 (PMC10239504; doi:10.1038/s41467-023-38926-3)
Supplement: Supplementary file 5 — Solar Cells Reporting Summary [file 41467_2023_38926_MOESM5_ESM.pdf]

nature research

Corresponding author(s): Tianshi Qin, Wei Huang

Solar Cells Reporting Summary

Nature Research wishes to improve the reproducibility of the work that we publish. This form is intended for publication with all accepted papers reporting the characterization of photovoltaic devices and provides structure for consistency and transparency in reporting. Some list items might not apply to an individual manuscript, but all fields must be completed for clarity.

For further information on Nature Research policies, including our data availability policy, see Authors & Referees.

Experimental design

Please check: are the following details reported in the manuscript?

1. Dimensions

Area of the tested solar cells
- [x] Yes
- [ ] No

The general-area device is 0.1 cm^2. The larger-area device is 1 cm^2. The certificated device area is 8.925 mm^2.

Method used to determine the device area
- [x] Yes
- [ ] No

The metal mask.

2. Current-voltage characterization

Current density-voltage (J-V) plots in both forward and backward direction
- [x] Yes
- [ ] No

The (J-V) plots are provided in the manuscript.

Voltage scan conditions
For instance: scan direction, speed, dwell times
- [x] Yes
- [ ] No

The bias ranges is from -0.1 to 1.3 V under reverse and forward voltage scan. Speed:233 mV/s, Dwell time:0.1s.

Test environment
For instance: characterization temperature, in air or in glove box
- [ ] Yes
- [ ] No

All J-V results are measured at 25 ±2°C in glove box.

Explain why this information is not reported/not relevant.

Protocol for preconditioning of the device before its characterization
- [x] Yes
- [ ] No

No precondition is used in this work.

Stability of the J-V characteristic
Verified with time evolution of the maximum power point or with the photocurrent at maximum power point; see ref. 7 for details.
- [x] Yes
- [ ] No

Time evolution of the maximum power point and the photocurrent at maximum power point are provided in Supplementary Fig. 20 .

3. Hysteresis or any other unusual behaviour

Description of the unusual behaviour observed during the characterization
- [x] Yes
- [ ] No

Very minor hysteresis for the devices.

Related experimental data
- [x] Yes
- [ ] No

J-V plots under reverse and forward are provided in Figure 4a.

4. Efficiency

External quantum efficiency (EQE) or incident photons to current efficiency (IPCE)
- [x] Yes
- [ ] No

IPCE are provided in Fig. 4b in the manuscript.

A comparison between the integrated response under the standard reference spectrum and the response measure under the simulator
- [x] Yes
- [ ] No

IPCE spectra demonstrated matchable integrated JSC values (<5% deviation) to J-V scan data.

For tandem solar cells, the bias illumination and bias voltage used for each subcell
- [ ] Yes
- [x] No

Tandem solar cells are not covered in this paper.

5. Calibration

Light source and reference cell or sensor used for the characterization
- [x] Yes
- [ ] No

Current density-voltage (J–V) curves were measured using a solar simulator (Class 3A, XES-40S3, SAN-EI) at AM1.5G illumination equipped with a Keithley 2400 source meter. The standard silicon solar cell calibrated by Newport was used to calibrate the light intensity to AM1.5G one sun (100 mW cm^-2).

Confirmation that the reference cell was calibrated and certified	☒ Yes ☐ No	The standard silicon solar cell calibrated by Newport was used to calibrate the light intensity to AM1.5G one sun (100 mW cm^-2).
Calculation of spectral mismatch between the reference cell and the devices under test	☐ Yes ☒ No	The standard silicon solar cell calibrated by Newport was used to calibrate the light intensity to AM1.5G one sun (100 mW cm^-2).

6. Mask/aperture

Size of the mask/aperture used during testing	☒ Yes ☐ No	Metal mask in general measurement is with area of 0.1 cm^2. The metal mask area of larger PSC is 1 cm^2. The certificated metal mask area is 8.925 mm^2.
Variation of the measured short-circuit current density with the mask/aperture area	☐ Yes ☒ No	All J-V results are measured with mask.

7. Performance certification

Identity of the independent certification laboratory that confirmed the photovoltaic performance	☒ Yes ☐ No	Certification report provided by Photovoltaic and wind power systems quality test center, Chinese academy of sciences for certification. (Test report No. PWQC-WT-P21110821-1R)
A copy of any certificate(s)<br>*Provide in Supplementary Information*	☒ Yes ☐ No	The certificates are provided in Supplementary Fig. 17 and 18.

8. Statistics

Number of solar cells tested	☒ Yes ☐ No	27 solar cells were tested.
Statistical analysis of the device performance	☒ Yes ☐ No	This has been stated in Supplementary Fig. 21.

9. Long-term stability analysis

Type of analysis, bias conditions and environmental conditions<br>*For instance: illumination type, temperature, atmosphere humidity, encapsulation method, preconditioning temperature*	☒ Yes ☐ No	Figure 4d, Normalized PCE of encapsulated PSCs according to ISOS-L-2 protocol (1-sun illumination and 65°C, in N2 atmosphere). Figure 4e, Normalized PCE of encapsulated PSCs according to the ISOS-D-3 protocol (85% RH and 85°C, in damp-heat chamber). All error bars represent the standard deviation of six devices. Figure 4f, Screenshots of unencapsulated control and perovskite/NVP films in water steam test (100% RH and 100°C) captured from Movie S1.
